# A 3D model to evaluate cell chemotaxis within a heterogenic tumor microenvironment

**DOI:** 10.1039/d5lc00763a

**Published:** 2026-01-20

**Authors:** Daniel B. Rodrigues, Daniela Cruz-Moreira, Luca Gasperini, Mariana Jarnalo, Ricardo Horta, Rui L. Reis, Rogério P. Pirraco

**Affiliations:** a 3B's Research Group, I3Bs – Research Institute on Biomaterials, Biodegradables and Biomimetics, University of Minho, Headquarters of the European Institute of Excellence on Tissue Engineering and Regenerative Medicine, AvePark Parque de Ciência e Tecnologia Rua Ave 1, Edifício 1 (Sede), 4805-694, Barco Guimarães Portugal rpirraco@i3bs.uminho.pt; b ICVS/3B's – PT Government Associate Laboratory Braga/Guimarães 4805-017 Portugal; c Department of Plastic and Reconstructive Surgery, and Burn Unit, Centro Hospitalar de São João Porto Portugal; d Faculty of Medicine, University of Porto Portugal

## Abstract

As studies continue to bring forward data on both the complexity and heterogeneity behind the tumor microenvironment, new strategies to understand and unravel the cellular interactions that regulate tumor progression and tumor cell invasion are required. Here, we present a novel and tailorable 4-well 3D culture chamber design capable of studying chemotaxis between several distinct cell types and a cancer cell population of interest. The use of a type I collagen hydrogel as the 3D substrate allowed for a differential molecule diffusion, in which rate of diffusion was associated with molecular weight. When culturing different human stromal cells (hASCs, hDMECs and hDFbs) in the outer wells while keeping VMM15 melanoma cells within the central well it was observed that hASCs and hDFbs presented directional migration throughout the collagen matrix towards the tumor cells. Further analysis revealed a higher area of migration present in the hDFbs when compared to the hASCs, supporting the potential of this system to study the recruitment of supporting cells by cancer cells and how this may impact tumor invasion.

## Abstract 2

Custom tailored 3D chemotaxis chambers allow better comprehension of intercellular crosstalk between tumor and stromal cells corroborating the role of tumor cells in the recruitment of supporting cells into the tumor microenvironment.

## Graphical abstract

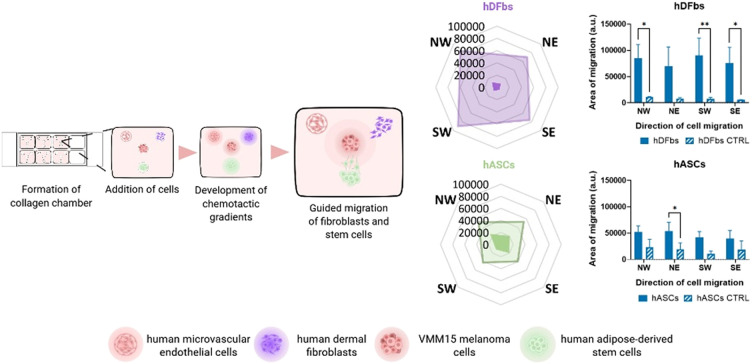

## 1. Introduction

Cancer remains a major burden in modern society, with close to 1.3 million deaths predicted in Europe alone for the year of 2024.^1^ This drives the need to better understand all aspects of tumor behavior, such as the interactions that take place within the tumor microenvironment (TME), in order to develop novel therapeutics with higher efficacy. One way to achieve this is the use of more biosimilar tumor models that better recapitulate the interactions happening within native tumors, allowing a more accurate prediction of antitumor drug response.

The TME is a highly heterogeneous milieu, comprised of tumor cells and additional cells that can include stromal cells such as endothelial cells (ECs), cancer-associated fibroblasts (CAFs), mesenchymal stem cells (MSC), pericytes and immune cells like tumor-associated macrophages and lymphocytes. Cell–cell interactions between these cells have been described^2–4^ and act as a major driving force promoting tumor progression.^4,5^ Additionally, cell–extracellular matrix (ECM) interactions are also key to regulate tumor growth and metastasis. These interactions are mediated by a variety of different factors such as chemokines, adhesion molecules, cytokines, growth factors or even matrix metalloproteinases (MMPs), eliciting responses not only at the cellular level but also modulating both the structural and biochemical properties of the ECM. A deregulation of certain pathways, such as selectin and epidermal growth factor-mediated signaling pathways, has been found to promote tumor growth and metastasis, along with altered expression levels of MMPs.^6–8^

It has been reported that tumor cell invasion, migration and dissemination are most efficient when occurring in a directed manner.^9^ This directed cell migration may be in response to several different forms of stimuli such as chemotatic, haptotactic, electrostatic and durotatic.^10^ Chemotaxis is defined as the phenomenon by which movement is directed in response to an extracellular chemical gradient and has been shown to favor tumor cell dissemination as reviewed elsewhere,^11^ while haptotaxis is directional cell movement in response to a gradient of adhesion such as to ECM or substrate-bound chemoattractants.^12^ In the specific case of melanoma, it has been shown that, for instance, IL-8 expression by endothelial cells possesses chemotactic activity towards WM35 melanoma cells, mediated by the CXC-chemokine receptor CXCR1.^13^ Other studies have associated the outward migration of melanoma cells from within the TME with self-generated lysophosphatidic acid (LPA) gradients. Cells create their own gradients by acting as a sink, breaking down locally present LPA, and thus forming a gradient that is low in the tumor and high in the surrounding areas allowing cells to migrate throughout this outward-directed gradient.^14,15^ In other works, ECM molecules laminin, fibronectin, and type IV collagen have been associated to both chemotaxis and haptotaxis of the A2058 human melanoma cell line, depending on whether the ligand was in solution or bound to a substrate.^16^ Additionally, vitronectin has also been implicated in chemotaxis and haptotaxis in A2058 human melanoma cells, mediated by integrin α_v_β_3_.^17^ These aforementioned studies, while contributing with worthwhile findings, are still based on 2D approaches which lack the complexity observed in the TME from its 3D nature to its cellular heterogeneity. Therefore, there is a pressing need for models better representing that native complexity to allow a more comprehensive understanding of cell behavior and cell communication.

Considering the limited variety of systems available to study the heterogeneity of the TME and its complex web of cell interactions, namely that of melanoma that is considered as one of the most heterogeneous,^18^ a type I collagen-based 3D culture system was developed. This system comprised 4 discrete compartments, three encompassing different stromal cells typically found in the melanoma TME,^19^ equidistant to a central compartment housing melanoma cells.

For operational consistency, cells were seeded into 1.2 mm-diameter wells patterned within the collagen matrix. This configuration enabled the establishment of chemotactic gradients between compartments, thereby driving directed migration of cells into the surrounding 3D hydrogel. Resultant migratory dynamics were subsequently quantified.

## 2. Experimental section

### 2.1 Design and fabrication of master pattern *via* direct micromachining

**Fig. 1:**
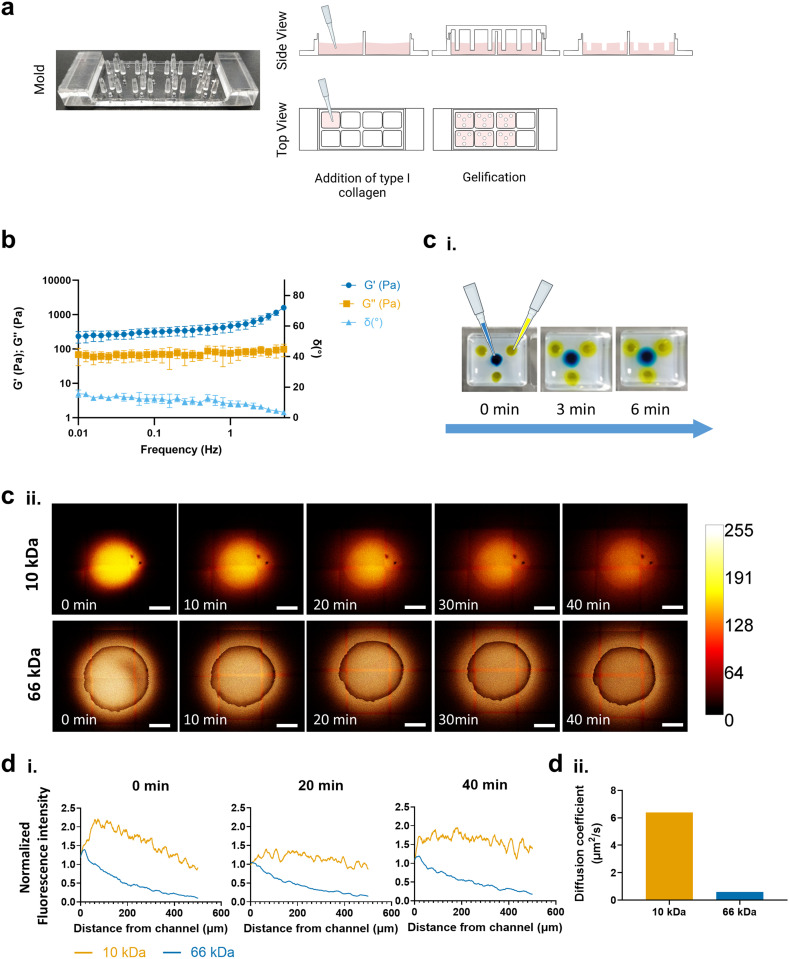
Chemotaxis chamber design and characterization. (a) Schematics of the development of collagen chemotaxis chambers. (b) Storage and loss moduli, as well as phase angle, as function of frequency for collagen gel (4 mg ml^−1^) (*T* = 37 °C). (ci) Yellow and blue dyes were injected into the wells. A merge of both colors was observed at the interface between the diffusing solutions. (cii) Fluorescent image sequence of both albumin–fluorescein isothiocyanate conjugate and Rhodamine B isothiocyanate–dextran wt ∼10 000 Da in the chemotaxis chamber at 0, 10, 20, 30 and 40 minutes respectively. (di) Diffusion (fluorescence) as a function of distance throughout the hydrogels was determined as the absolute fluorescence intensity normalized to the intensity at *x* = 0 for each time point. (dii) Diffusion coefficients calculated by fitting diffusion profile curves to a diffusion equation. Original magnification, scale bar = 500 μm.

A type I collagen chemotaxis chamber was fabricated by hydrogel molding and integrated into μ-Slide culture chambers to facilitate handling and live microscopic imaging. The chamber design is illustrated in Fig. 1 and the corresponding mold schematic is provided in Fig. S1. A reusable master mold was employed as a cover for the μ-Slide, imprinting the collagen hydrogel during gelation to define the microarchitecture. The master mold was designed to generate eight independent chambers, each consisting of a central well positioned equidistantly from three peripheral wells, which were themselves evenly spaced. The mold has an overall footprint of 75 mm by 25 mm and a height of 1 cm, with individual chambers measuring 10.65 mm by 9.4 mm. Cylindrical pillars (1.2 mm diameter each, 7 mm high) defined the microwell features. The mold layout was designed using computer-assisted design (CAD) software (Fusion 360, Autodesk Inc.). Master molds were fabricated from polymethyl methacrylate (PMMA) by direct micromachining (MDX-50, Roland DG) using a ZHS-100 tool operated at 15 000 rpm and a feed rate of 480 mm min^−1^. Machining consisted of sequential roughing and finishing steps to ensure high feature fidelity. Features of the pattern were sharply contoured to enable good pattern resolution. Following fabrication, the PMMA molds were cleaned, surface polished by brief exposure to dichloromethane vapor and sterilized by UV radiation. Upon imprinting into the collagen matrix, the pillars formed stable microwells suitable for three-dimensional cell culture and chemotaxis assays.

### 2.2 Hydrogel production and characterization

Type I collagen from bovine skin (Sigma) was prepared according to the manufacturer's recommendations. All components were maintained on ice during preparation. Briefly, 10× PBS was combined with 1 N NaOH to adjust pH, followed by the addition of sterile ice-cold water to obtain a final collagen concentration of 4 mg mL^−1^. The appropriate volume of type I collagen stock solution (6 mg mL^−1^) was then added, and the mixture was gently homogenized and maintained on ice.

Prior to casting, the collagen solution was degassed under vacuum to remove entrapped air bubbles, using repeated 20 s cycles until cavitation was no longer observed. Evaporation during degassing was minimized and monitored. Subsequently, 400 μL of collagen solution was dispensed into each well of μ-Slide 8-well chambers (Ibidi), and the chemotaxis mold was carefully positioned on top. Gelation was induced by incubating the assemblies at 37 °C for 2 h.

Rheological characterization of the resulting collagen hydrogels was performed on a rheometer (Malvern Kinexus Pro+). For the derivation of storage and loss moduli, hydrogels were loaded into the rheometer, and a stainless-steel geometry (8 mm of diameter and 4°) was lowered until a minimal normal force (0.05 N) indicated contact. The linear viscoelastic region (LVER) for the hydrogel chambers was then determined through a strain amplitude sweep test (from 0.01 to 10% strain amplitude), at a constant shear rate (1 Hz). A fixed strain within that region was then selected to obtain the frequency sweep curves (from 0.1 to 10 Hz) and then derive the storage (*G*′) and loss (*G*″) shear moduli profile of the hydrogels. Data represents the mean of three independent experiments (*n* = 3), with all measurements performed within the linear viscoelastic regime. Reported moduli correspond to values at 1 Hz values. The loss tangent was calculated as tan *δ* = *G*″/*G*′. The storage Young's modulus at the same frequency was estimated assuming linear isotropy and a Poisson's ratio *ν* : *E*′(*ω*) = 2*G*′(*ω*)(1 + *ν*). We assumed *ν* = 0.5 to reflect near-incompressibility^20,21^ of hydrated collagen networks at 1 Hz. Uncertainties in *E*′ were propagated from the variability in *G*′.

### 2.3 Characterization of diffusion

To qualitatively assess molecular transport within the chemotaxis chambers, food dyes were simultaneously introduced into the outer wells (yellow) and the central well (blue), and color diffusion was monitored over time. For quantitative characterization of solution gradients within the developed system, 10 μM solutions of both albumin–fluorescein isothiocyanate conjugate (Sigma) and Rhodamine B isothiocyanate–dextran wt ∼10 000 (Sigma) were prepared in PBS and 2.5 μl were added to each well to be analyzed.

Fluorescence diffusion throughout the gels were observed under an SP8 Leica laser confocal microscope (Leica Microsystems, Wetzlar, Germany). Time-lapse images were taken at 0 min, 10 min, 20 min, 30 min and 40 min. The fluorescent intensities were quantified using Leica Application Suite X ver. 3.7.2.23383 (Leica Microsystems). Briefly, for each well, 4 different line profiles of 500 μm were drawn from the most outer edge of each axes (north, south, east and west) outwards into the gel. The fluorescent intensities were recorded throughout each ROI and an average in function of distance was calculated per well. These intensity curves were then fit to a one-dimensional diffusion equation with a point source:1
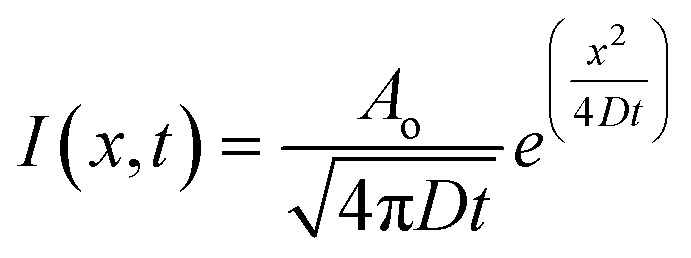
where *I*(*x*, *t*) was the intensity at each time position, *A*_o_ was the total amount of diffusing substance, *D* was the diffusion coefficient, *t* was the time following the injection of the fluorescent probes, and *x* was the radial distance from the center. Fits were performed using the full spatial and temporal datasets for each fluorescent probe, and the extracted diffusion coefficients represent the best-fit values obtained from the averaged experimental profiles.

### 2.4 Cell lines and culture conditions

hTERT Dermal Microvascular Endothelial Cell, Neonatal (ATCC® CRL-4060™) were obtained from ATCC and cultured in EGM-2 MV Microvascular Endothelial Cell Growth Medium-2 BulletKit (Lonza, USA). Human dermal fibroblasts (hDFbs) and human adipose derived stromal/stem cells (hASCs) were harvested from human skin samples obtained from abdominoplasties. Both hDFbs and hASCs were cultured in α-MEM, supplemented with 10% fetal bovine serum (FBS, Invitrogen, USA) and 1% antibiotic/antimycotic (Sigma, USA). Human cell line VMM15 (ATCC® CRL-3227™), derived from metastatic melanoma lesions, were grown as adherent cultures in RPMI 1640 Glutamax supplemented with, 50 μM 2-mercaptoethanol (Gibco, Scotland, UK) 10% fetal bovine serum (FBS) and 1% antibiotic/antimycotic (Sigma, USA). All cells were kept in culture at 37 °C in a humidified atmosphere under 5% CO_2_.

### 2.5 Seeding of cells on collagen gels

To determine the optimal seeding density for use in the collagen-based chemotaxis chambers, cells were seeded at four densities (1 × 10^3^, 5 × 10^3^, 10 × 10^3^ and 15 × 10^3^) in a total volume of 2.5 μL per well. Cell were left to adhere to the bottom of the well, on top of the collagen substrate, for 1 h at 37 °C, after which 175 μl of EGM-2MV culture medium was added to the whole chamber. Cell confluency was followed overtime through bright-field microscopy (Axio Observer inverted microscope, Zeiss, Germany).

Cell confluency was quantified from brightfield images (1496 × 1495 pixels) using Fiji (ImageJ, version 1.54p). Images were converted to 8-bit, background-subtracted (rolling ball radius = 6 px, light background), and contrast-enhanced (0.35% saturation, normalized). Segmentation was performed with the Trainable Weka Segmentation plugin, and probability maps were thresholded using the Yen algorithm. Confluency was calculated as the area fraction of cell-covered regions relative to the total image area. The cell density that yielded a suitable confluency to allow for cell growth was chosen to carry out the remaining work.

To evaluate the impact of different chemotactic gradients produced by stromal cells on VMM15 tumor cell invasion, cells were seeded within the chemotactic chamber such that the VMM15 tumor cells seeded within the center chamber would be subjected to all three of the chemotactic gradients formed by the hDMECs, hDFbs and hASCs.

### 2.6 Cell migration and cell tracking

Considering the preliminary titration that was performed to determine the optimal cell densities, a density of 1 × 10^4^ cells per well was selected and used for all subsequent experiments. Cell migration was followed over time on an Axio Observer inverted microscope with incubation (37 °C, 5% of CO_2_), and Zen 3.2 (blue edition) software (Zeiss, Germany) at every 30 minutes intervals for 3 days.

For image analysis, brightfield images were first cropped using ImageMagick 6.9.11 to obtain a 1 : 1 ratio image centered on each well for each time point of the experiment. Central spherical region was excluded to isolate migrating cells, and each image was divided into four equal quadrants corresponding to intercardinal directions (NW, NE, SW and SE). Pixel classification was performed using Ilastik (pixel classification workflow) to generate probability maps distinguishing foreground (cells) from background. These grayscale probability maps were then imported into CellProfiler 2 for segmentation and quantification of the area occupied by migrating cells. Given the binary nature of the probability maps, manual thresholding was applied. This approach is appropriate when the input image displays a stable or negligible background, as was the case in the present dataset. A threshold value of 0.5, corresponding to foreground and background values of 1 and 0 respectively, was used to delineate the objects. All image processing and analyses were performed on a workstation equipped with a 7th generation Intel® Core™ i7-7500U processor (2.7–3.5 GHz), 16 GB DDR4 memory (2133 MHz), and Intel HD Graphics 620.

### 2.7 Immunocytochemistry

After cell migration assays, gels were fixed in 4% paraformaldehyde solution (Pierce, USA) at 4 °C for 48 h. For immunocytochemistry, samples were permeabilized with 0.2% Triton X-100 and unspecific staining was blocked with 3% bovine serum albumin (BSA; Sigma-Aldrich). Subsequently, the gels were incubated in PBS with 0.2% Triton X-100 (Sigma), fluorescein-conjugated phalloidin (1 : 250; Invitrogen, Netherlands) and 1 : 1000 DAPI (1 : 1000; Biotium, USA). Samples were examined under a Zeiss LSM980 AiryScan 2 Confocal Microscope, Zen 3.6 (blue edition) software (Zeiss, Germany).

### 2.8 Statistical analysis

Data are expressed as mean ± standard deviation of three independent experiments. Data normality was assessed using Shapiro–Wilk test prior to statistical testing. Statistical analysis was performed using one-way or two-way analysis of variance (ANOVA), as appropriate, followed by either a Tukey's or Bonferroni's multiple comparison *post hoc* test. Significance was set to 0.05 (95% of confidence interval). Significance levels were analyzed with GraphPad Prism statistical software v8.0 (GraphPad Software, USA) and indicated as *p* values (*p < 0.05, **p < 0.01, and ***p < 0.001).

## 3. Results

### 3.1 Characterization of type I collagen chemotactic chambers

To create a 3D chemotaxis chamber, a master mold was designed and conceived to be compatible with commercially available 8-well slides (Ibidi) (Fig. 1a). The central pillar of the mold was designed to create a well to accommodate tumor cells, whereas the three remaining pillars, equidistant to the center pillar, would create three wells to host stromal cells. The mold was produced by microdrilling a 75 mm × 25 mm × 10 mm PMMA piece in which the obtained pillars presented an average height of 6.95 ± 0.05 mm and an average diameter of 1.63 ± 0.05 mm, with the external pillars being equally spaced 1.46 mm from the central pillar. This distance between wells was chosen to favor interactions between cells in the outer wells and cells in the center well over interactions between cells in the three outer wells. Furthermore, that distance is within an acceptable range considering the formation of chemotactic gradients according to several reports.^22–24^ Within the mold design, an additional hole was placed in each chamber to prevent hydrogel overflow. Having obtained the master mold required to produce the chemotaxis chambers, the fabricated hydrogels were characterized before validating their use to study chemotaxis.

While the elastic nature of cancer tissues has already been described,^25,26^ biological components, such as the extracellular matrix (ECM), also possess viscoelastic responses to varying stimuli. Viscoelasticity, as the term implies, is the property of a material that exhibits mechanical traits of both elastic solids and viscous fluids. This viscoelastic behavior of both ECM and cancer cells has been shown to play a crucial role in the progression of cancer.^27,28^ Considering this, we assessed the rheological properties of the developed 3D chemotaxis chambers. At 1 Hz, 4 mg mL^−1^ collagen I hydrogels showed *G*′ = 460.78 ± 141.09 Pa and *G*″ = 74.69 ± 32.96 Pa (Fig. 1b). The loss tangent was tan *δ* ≈ 0.162, indicating predominantly elastic response at this frequency. Using *ν* = 0.5, *E*′(1 Hz) = 3*G*′ = 1.38 ± 0.42 kPa. This value is frequency-dependent and represents dynamic stiffness rather than the quasi-static modulus.

To determine whether the developed collagen hydrogels would be suitable for the formation of chemotactic gradients, we assessed diffusion throughout the gels. Firstly, two dyes, yellow (outer wells) and blue (center well) were injected into the wells, in which over time, the color in the wells changes concentrically from distinct yellow (outer wells) and blue (inner well) to a green tint where both dyes meet (Fig. 1ci) suggesting that the produced collagen hydrogel allows for diffusion. To further characterized diffusion properties, we have studied the gradient profiles generated by two model molecules, namely albumin–FITC (66 kDa) and Rhodamine B–dextran (10 kDa). These two tracers were selected for their similarity in molecular weight (MW) to known biological molecules that interact with cells through gradient-based signaling [*e.g.*, interleukin-8 (IL-8, 8.6 kDa),^29^ IL-2 (15 kDa),^30^ tumor necrosis factor (TNF, 17 kDa),^31^ fibroblast growth factor 2 (FGF2, 18 kDa),^32^ vascular endothelial growth factor (VEGF, 45 kDA)^33^]. Fluorescent intensities in the chamber were measured over time and compared (Fig. 1cii). Even after 40 minutes, albumin displayed a higher fluorescent intensity closest to the well, indicative of lower diffusibility in the collagen chambers. In contrast, dextran enabled increased diffusion, as evidenced by the equalization of fluorescence levels across the hydrogel over time.

The one-dimensional diffusion equation (eqn (1)) was then fit to the data from Fig. 1di. The resulting diffusion coefficients (*D*) for each of the fluorescent probes are shown in Fig. 1dii. As for Rhodamine B–dextran (10 kDa), the diffusion coefficient was determined to be 6.393 μm^2^ s^−1^, while albumin–FITC (66 kDa) presented a coefficient of 0.6009 μm^2^ s^−1^. This agrees well with visual inspection of the diffusion behavior in Fig. 1cii and the anticipated effects of molecule size on this parameter.

These results confirm the potential of the developed collagen chamber to be used as a system to study chemotactic cell–cell interactions.

### 3.2 Cell migration in response to chemotactic gradients

Chemotaxis has long been described to play a crucial role in tumor progression and in directed migration of cells within the TME. These migration patterns have been shown to be dependent on the type of cancer and the surrounding factors within the TME. Previous reports have suggested that stromal cells may lead the tumor invasive front, opening channels within the stroma that allow for the migration of tumor cells.

**Fig. 2:**
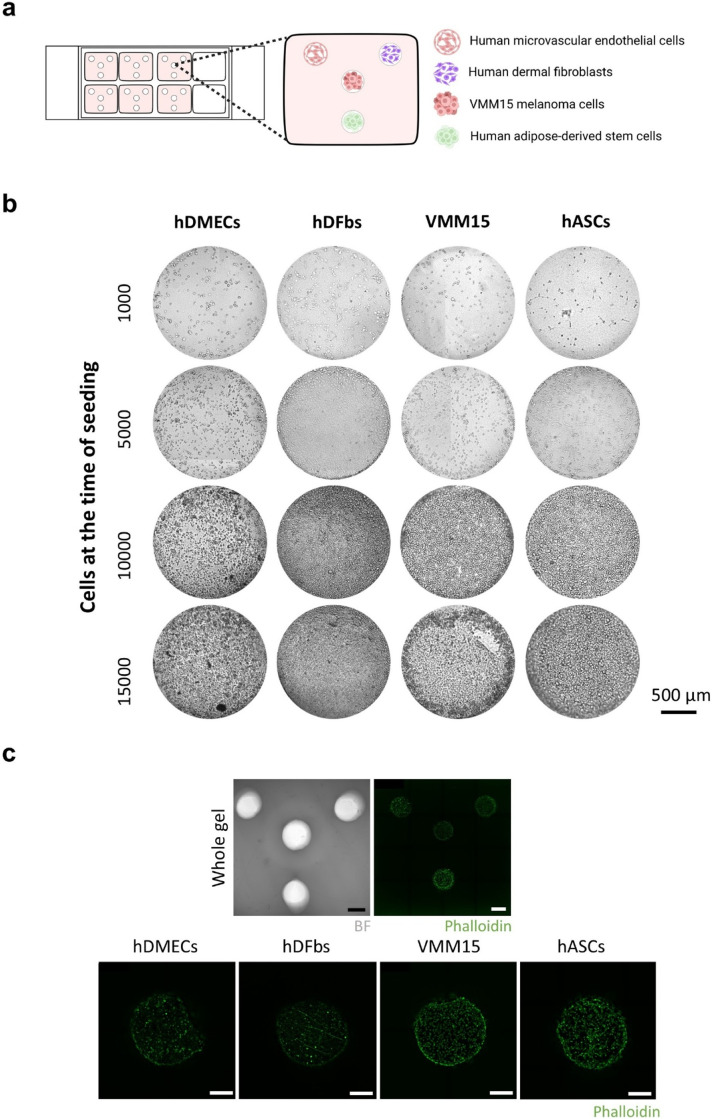
Chemotaxis chamber design and characterization. (a) Schematics representation of VMM15, hDMECs, hASCs and hDFbs cell organization within the collagen chemotaxis chambers. (b) Phase-contrast images of cells after 2 h of seeding in the collagen chemotaxis chambers at increasing cell densities as described, scale bar = 500 μm. (c) Morphological analysis of cells, 24 h after seeding, by FITC-phalloidin (green = phalloidin). Original magnification of whole gel, scale bar = 1 mm. Original magnification of individual wells, scale bar = 400 μm.

To test the developed chambers, cells were seeded in the wells to assess the putative effects of cellular crosstalk between tumor cells and stromal cells on migration patterns. Chemotactic gradients were expected to form and regulate cell migration directionality within the chamber. VMM15 melanoma cells were placed in the center chamber in order to influence all of the surrounding cells equally, while also being under the effect of the signals secreted by the stromal cells (Fig. 2a). In the outer chamber wells, human dermal microvascular endothelial cells (hDMECs), human adipose derived stromal/stem cells (hASCs) and human dermal fibroblasts (hDFbs) were seeded. A titration assay was first carried out to understand the correct cellular confluency required for the gels (Fig. 2b). Four different densities of 1 × 10^3^, 5 × 10^3^, 10 × 10^3^ and 15 × 10^3^ cells per well were tested. It was observed that the density of 15 × 10^3^ cells per well led to cell aggregation and detachment with increasing time of culture. Cellular confluency (Fig. S2) was quantified, and a seeding density of 10 × 10^3^ cells per well was selected, corresponding to an average confluency of approximately 83% across the four cell types examined. FITC-phalloidin labelling was performed to confirm cell attachment through morphology assessment (Fig. 2c).

**Fig. 3:**
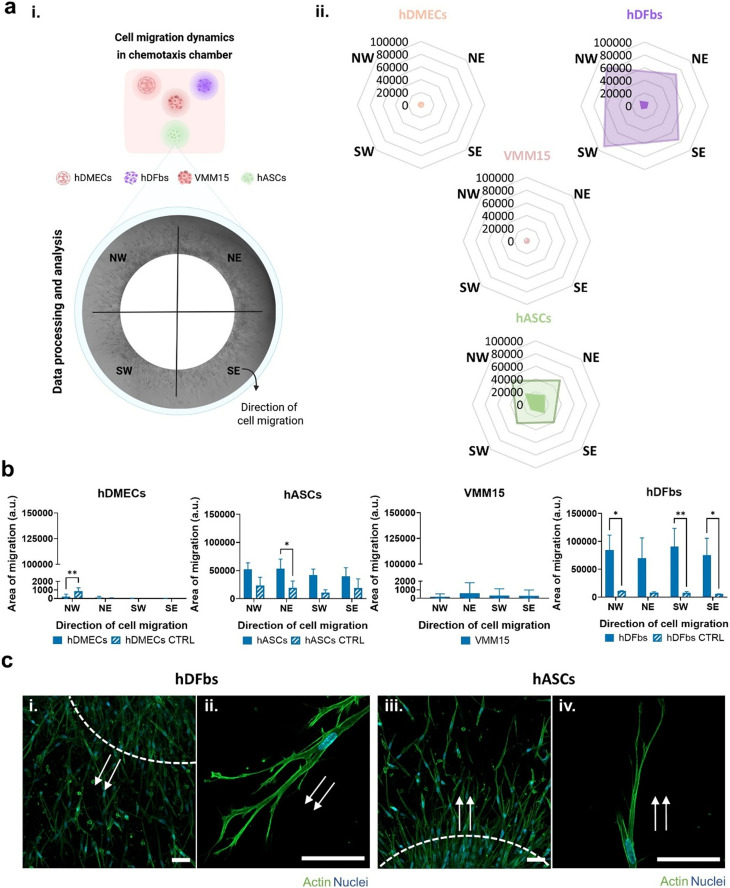
Chemotaxis of stromal cells in a 3D tumor microenvironment model. (ai) Schematics of cell organization within the collagen chemotaxis chambers. (aii) Spider charts depicting tumor cell driven chemotaxis of stromal cells. (b) Quantification of the migration profile of hDMECs, hDFbs, VMM15 and hASCs after time-lapse microscopy. (ci–iv) Double-labeling showing the distribution of F-actin (green) and cell nuclei (blue). Arrows in white demonstrate the direction of cell migration. Scale bar = 50 μm.

Observation of cell migration in these chambers by time-lapse microscopy was performed. Segmentation of the adjacent area to the culture wells showed that hDMECs and VMM15 melanoma cells lacked any form of migration. hASCs and hDFbs, on the other hand, presented clear evidence of directional migration throughout the type I collagen chamber (Fig. 3aii). Characterization of directional migration was attained by quantifying the area occupied by cell migration in a 4-axis template (Fig. 3ai) consisting in NW, NE, SW and SE quadrants. An increased area of migration in the direction of the VMM15 tumor cells was shown for hDFbs, represented by a significant shift towards the NW (*p* < 0.05), SW (*p* < 0.01) and SE (*p* < 0.05) quadrants when compared to the control condition lacking VMM15 cells within the central chamber. Regarding hASCs, an increased migration towards the VMM15 tumor cell well was also observed, reflected by a larger area of migration towards the NE quadrant (*p* < 0.05) when compared to the control group (Fig. 3b).

Considering the directed migration that hASCs and hDFbs were undergoing, we considered relevant to evaluate cell morphogenesis during the observed migration. The spindle shape characteristic of a mesenchymal migration type was observed in cells, followed by an extension of lamellipodia and filopodia and the attachment of the protrusions to the surface (Fig. 3cii and iv). As clearly observed in the invading edge of the fibroblast condition (Fig. 3cii), a higher density of actin within the cell cytoskeleton correlates with the directionality towards the VMM15 cell chamber, similarly to hASCs (Fig. 3civ).

## 4. Discussion and conclusion

The TME is composed of a complex milieu of cells, including tumor cells, immune cells (DCs, macrophages, T cells, B cells, NK cells, neutrophils, and MDSCs), and stromal cells (CAFs, MSCs, tumor-associated adipocytes, tumor endothelial cells, and pericytes),^1^ which are in constant crosstalk and contact with one another. Chemotaxis is a mechanism that has long been described as playing a critical role in tumor progression. By definition, it is the directed movement of cells in response to an extracellular chemical gradient and has been associated to several different types of cancer.^11^ Hence, it is a critical feature to be modelled in 3D culture systems that study cancer biology. Here we present a system that allows the discretization of particular cell–cell interactions due to the geographical separation of up to 4 different cell populations. While it can be viewed as merely an improved two-well system, since all interactions are happening simultaneously, the resemblance to the native scenario is improved non-linearly, arguably providing a much better model to study intercellular crosstalk.

Similar strategies have been employed over time with the aim of creating more accurate 3D cell culture assays for studying intercellular communication and cell migration. These include approaches that allow direct cell confrontation in 3D,^34^ simple 3D hydrogels systems,^35^ transwell-based assays,^36–38^ the use of commercial chemotaxis chips^39,40^ or custom-built microfluidics systems.^41,42^ While traditional transwell systems are simple, low-cost, and allow for high throughput, they are many times incompatible with real-time imaging. On the other hand, commercial chemotaxis chips, such as those commercialized by IBIDI®, are simple and standardized and allow for controlled gradient generation and real-time imaging; however, they lack throughput and the ability to support multicellular assays. Microfluidic systems address some of these limitations, as they allow co-cultures, are compatible with gradient generation, enable real-time imaging, and preserve cell phenotype. Nevertheless, they mostly present planar geometries, make the collection of cell migration data challenging, and require specialized equipment for chip fabrication. The system we propose here, while simple, complements existing technologies by building on 3D hydrogels as a means to study cell migration in response to biochemical cues. In addition to the simplicity and low cost of these systems, we demonstrate that, through simple fabrication techniques, it is possible to mimic key multicellular interactions found in the TME in real time. These interactions can be analyzed using a straightforward image analysis workflow, while also enabling the study of multiple cell–cell interactions within a single assay.

While several chemotaxis systems rely on a distance of guidance in response to a chemotactic molecule in the micrometer order of magnitude,^24,43,44^ a few studies have pointed to a cellular response towards shallow gradients. The minimum concentration difference that is required across motile cells for directional sensing is reported to be around 1–2%,^45,46^ where cells like neutrophils have shown even greater levels of sensitivity as low as 0.5%.^47,48^ It has been demonstrated that concentration differences as low as 0.1% can guide axonal growth, with a single exponential gradient supporting navigation over distances of up to approximately 2 cm.^49^ More recently, Tweedy *et al.* showed that self-generated chemotaxis can operate over large spatial and temporal scales, considering that these gradients are local and mobile within the field. In their study, directed chemotactic movement was maintained for up to 7 hours over a distance of 5 mm.^50^ In fact, increased attention is being given to these types of gradients since they appear as strong candidates to explain cell migration over long distances such as between tissues (*e.g.*, metastization). We propose that such a mechanism may contribute to the migration observed in our system and, as a consequence, our system emerges as a suitable platform for studying this phenomenon.

Our developed chemotaxis chamber presents a distance of 1.46 mm from the most outer region of the center well to the outer region of either of the 3 adjacent wells, slightly above what has been classically considered as the upper limit for externally imposed chemotactic gradients but well within biological plausibility considering self-generated gradients. This, along with the higher distance between adjacent outer wells, favors the formation of chemotactic gradients between stromal cells and tumor cells, rather than between stromal cells alone. Moreover, Dravid *et al.* demonstrated through the development of a diffusion-based gradient generator that it is possible to generate diffusive concentration gradients with molecules whose molecular weights are similar to those of biological molecules over distances of several millimeters.^22^ Others have also shown this extensive range of biomolecule diffusion throughout gels in lengths above 2.5 mm.^23,24^

In this work, we used type I collagen for the development of the model given that collagen gels have been proven to serve as better mimic in terms of molecule diffusion for the development of new models,^51^ especially considering the high abundance of this protein in the extracellular matrix of tumors.^52^ While polysaccharides such as alginate, agarose, or gellan gum may be considered alternatives due to their similar viscoelastic properties, they lack cell adhesion sites like RGD sequences^53^ and possess a non-degradable nature, which limits their capacity to support cell migration in 3D structures.^54^

Our results show that the implemented chamber organization allowed molecule diffusion dependent on molecular weight, with a faster diffusion occurring for molecules of lower molecular weight. Knowing that the chosen molecular weights fall within the range corresponding to chemokines and cytokines, it can be assumed that molecules with a chemotactic role will be able to diffuse through the gel in a similar manner to the fluorescently tagged albumin and dextran. Additionally, since the mechanical properties of biomaterials impact cellular behavior,^55,56^ there is a need to meet physiologically relevant parameters. A study in a microfluidic platform showed that the migration speed of the cancer cells is influenced by the synergistic effect of channel stiffness and width, while also affecting mesenchymal-amoeboid transition.^57^ Indeed, the viscoelastic nature of the TME is ever clearer,^28,58^ which makes it of the upmost importance to take into consideration such features when modeling tumor cell behavior *in vitro*. Hydrogels tend to present a higher *G*′ while displaying a lower *G*″, indicative of the materials potential to store deformation energy with small dissipation, therefore behaving predominantly like solids.^59^ From the presented amplitude sweeps, not only was this behavior well evident as it also is representative of the behavior for a range of soft tissues.^60^ This viscoelastic nature is crucial to close the gap between *in vitro* tumor models and native tissue as it is one of the main driving forces for cancer tissue remodeling and tumor growth.^61^ Indeed, elevated fluid viscosity has been shown to allow for enhanced cancer cell migration and is tied to the metastatic potential of these tumors.^62^ Having our collagen hydrogel portray such mechanical behavior is crucial for its use as a system to study intercommunication between cells of the TME.

Given the demonstrated molecule permeability of the type I collagen matrix, the outer wells of the chamber were then populated with relevant stromal cells that would allow the study of their migratory response in the presence of tumor cells in the center well. While here the focus was essentially on cell migration driven by chemical gradients, chemotaxis, the effect of other mechanisms related with the properties of the 3D matrix, namely its stiffness (durotaxis)^63^ and fiber alignment (alignotaxis),^64,65^ cannot be ruled out although no stiffness or alignment gradient was implemented or otherwise detected.

A dynamic interplay is known to take place between tumor cells and stromal cells within and outside the TME. Different cell–cell interactions take place, leading to the recruitment by tumor cells of endogenous stromal cells into the TME^66,67^ or, conversely, to the migration of tumor cells following a chemotactic gradient set by noncancerous cells.^68,69^ Several different elements are known to play a part in this guided migration, namely cell adhesion molecules (CAMs), chemokines, cytokines and growth factors.^70–72^ Interplay with the ECM has also been shown to promote tumor progression and poor prognosis.^73,74^ An example of this are MMPs which are tied to the infiltration of cancer cells and metastasization.^6–8^

In the context of this work, directed migration of the tumor cells from the inner well outwards would be possible as suggested by studies showing that tumor cells are capable of creating local gradients of MMP-mediated degradation of ECM to drive their directed migration.^75–77^ However, it was the fibroblasts and the stem cells that migrated towards the well containing the tumor cells, showing the chemoattractant capacity of the latter towards stromal cells.^78–80^ These results clearly support that chemotactic gradients within the TME are orchestrated by tumor cells, dictating how stromal cells, such as fibroblasts and stem cells migrate. To gain a more comprehensive understanding of these results, additional analytical parameters, such as cell migration speed, directional persistence (indicating the degree to which a cell maintains its movement in a consistent direction rather than frequently changing course), and the chemotactic index, which measures how effectively a cell moves toward or away from the chemoattractant source, should be considered in future work.

To further support and confirm this conclusion, it is crucial to identify the key molecules responsible for this behavior in future studies, as well as to untangle the effect of intracellular communication. Omics technologies may enable deeper cellular-level analyses in future studies, particularly spatial proteomics and transcriptomics, which could reveal which cells sense chemotactic gradients and how they respond. Previous studies have identified several mediators of these gradients, including IL-8, which promotes migration and invasion of head and neck squamous cell carcinoma (HNSCC) cells through fibroblast-mediated extracellular matrix degradation.^81^ Transforming growth factor-β (TGF-β) also acts as a master regulator of stromal cell recruitment, activation, and tumor-stromal crosstalk. In prostate cancer, microenvironment-derived TGF-β1 recruits and activates mesenchymal stem cells (MSCs) into carcinoma-associated fibroblasts (CAFs).^82^ Additionally, basic fibroblast growth factor (bFGF), secreted by tumor cells, recruits bone marrow-derived mesenchymal stem cells (BMSCs) to the primary tumor site and promotes their differentiation into CAFs, thereby supporting tumor growth.^83^ Beyond biochemical cues that drive cell migration, mechanical-based signaling also plays an important role. Cells constantly deposit and remodel the surrounding matrix, leading to mechanisms such as durotaxis,^63^ topotaxis,^84^ or alignotaxis.^64,65^ While no mechanical characterization of the hydrogels was performed after cell culture, changes in mechanical stiffness and fiber density cannot be ruled out and may be important for maintaining an integrative view that combines different aspects of cell regulation. However, as the cells were exposed to the same concentration of collagen hydrogel in each chamber—an advantage of our system that provides a window into observing the behavior of different stromal cells simultaneously while minimizing exposure to hydrogel variability—we believe that, if such physical changes to the matrix occur, they are likely to be local and cell-driven rather than a side effect of the matrix properties adopted in our protocol.

While cell morphogenesis has always been linked to cell migration, a clear understanding on how cells undergo migration under chemotactic gradients has been missing. Filopodia distribution and their dynamics were shown to be dictated by the gradient of the Cxcl12a chemokine.^85^ While no such in-depth analysis was performed herein, it would be important to investigate a potential correlation between the observed directional migration and the VMM15 melanoma cell secretome. This analysis would be of high relevance in determining whether a similar behavior to that reported by the study above is reproduced. This could, in turn, open the path for novel therapeutics that could limit non-tumor cell recruitment to the microenvironment and therefore hamper tumor progression.

Here, we have explored engineering tools to present a co-culture device with novel features, namely the ability to assess the simultaneous directional migration of different stromal cell types in response to a stimulus from cancer cells, allowing for a better understanding of the intercellular interactions that drive tumor progression—more specifically, the selective recruitment of particular stromal populations by cancer cells. This aspect of the disease is often overlooked in more complex models that do not permit a clear evaluation of specific cellular interactions. Few, if any, devices allow for the selective evaluation of directed migration, let alone the simultaneous directional recruitment of multiple stromal types in response to a tumor cell population.

Although several distinct cell types critical to tumor progression and metastasis were considered in this study, it is important to acknowledge that the model remains an incomplete representation of the TME, as it lacks a key component, immune cells. These cells have been implicated in multiple essential processes, including extracellular matrix remodeling through the activity of MMPs, the establishment of immunosuppressive conditions that protect circulating tumor cells from immune surveillance, the promotion of angiogenesis, and the protection of cancer cells from cytotoxic immune responses.^86–89^

The formation of diffusion gradients was observed using model biomolecules and these were dependent on the molecular weight of the biomolecule. Upon seeding of stromal and melanoma cells in the different wells, differential migration of fibroblast and stem cells was observed towards the VMM15 melanoma cells, suggesting that chemotactic gradients were established, potentially mimicking intratumoral crosstalk between stromal and tumor cells. This demonstrates the potential of the developed chamber to be used as a tool to study and characterize mechanisms underlying tumor cell migration and metastasis.

## Conflicts of interest

The authors declare no competing interests.

## Supplementary Material

LC-026-D5LC00763A-s001

## Data Availability

The datasets generated and analyzed during this study are available from the corresponding author upon reasonable request. Image analysis pipelines have been included as part of the supplementary information (SI). Supplementary information is available. See DOI: https://doi.org/10.1039/d5lc00763a.
