# Supplementary material for: A 3D model to evaluate cell chemotaxis within a heterogenic tumor microenvironment
Source: Lab Chip. 2026 Jan 20;26(9):2747–60. doi: 10.1039/d5lc00763a (PMC12836168; doi:10.1039/d5lc00763a)
Supplement: LC-026-D5LC00763A-s001 [file LC-026-D5LC00763A-s001.pdf]

Supplementary Information (SI) for Lab on a Chip.
This journal is © The Royal Society of Chemistry 2026

# Supplementary Information

**A 3D model to evaluate cell chemotaxis within a heterogenic tumor microenvironment**

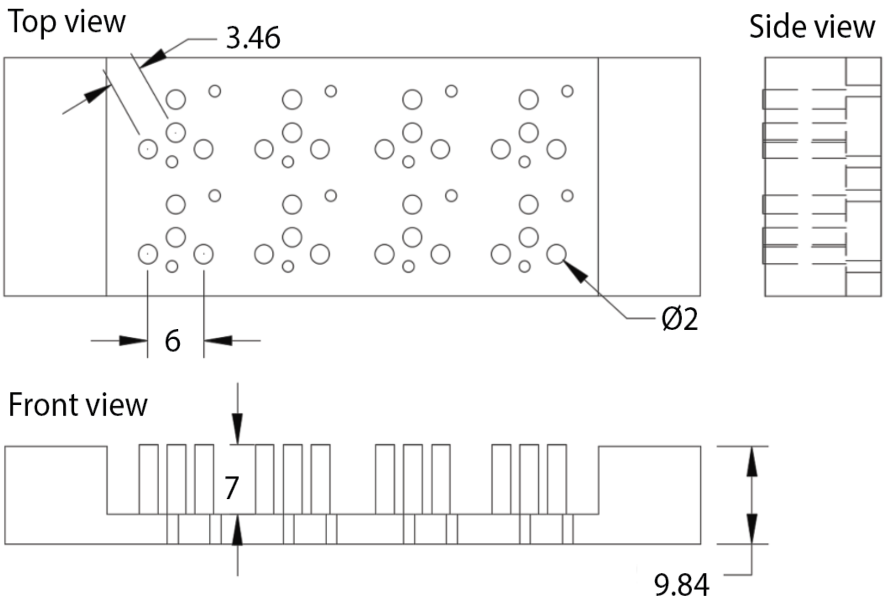


**Figure S1: Computer-aided design of chemotaxis chamber master mold.** 2-D view of top, side and front view of the master mold used to imprint the hydrogel chemotaxis chambers. Dimensaions are in mm.

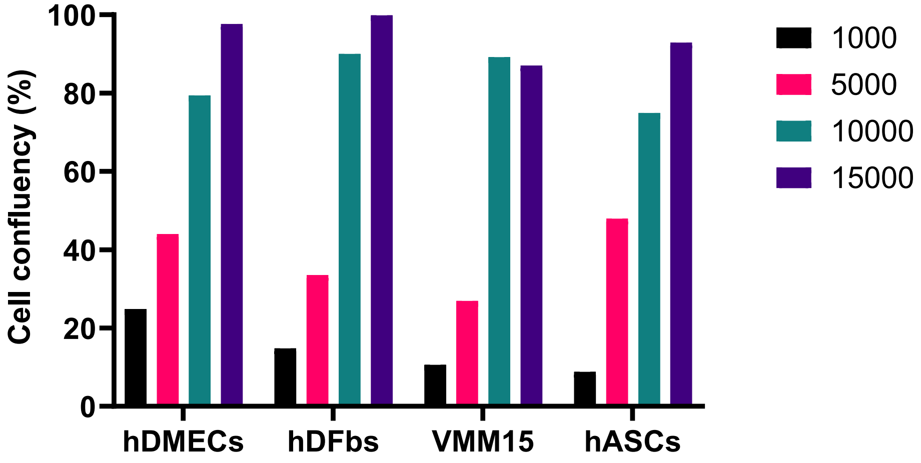


**Figure S2: Evaluation of cell confluency.** Cell confluency of hDMECs, hDFbs, VMM15 and hASCs 2h after seeding at densities of 1000, 5000, 10000 and 15000 per chamber well.

# 1. Image Classification and Segmentation (Illastik)

Original image
1538x 1118 (Pixels)

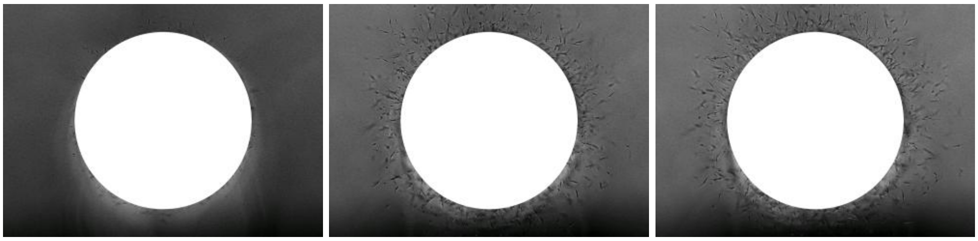

Pixel Classification with Ilastik

Classifier Training: Label 1 (Yellow) – Cells
Label 2 (Blue) - Hydrogels

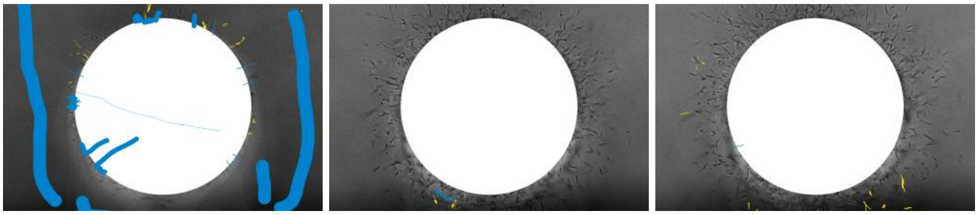

Probability Maps

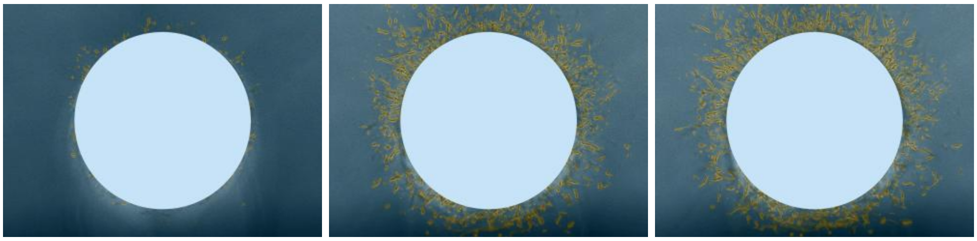

Simple Segmentation

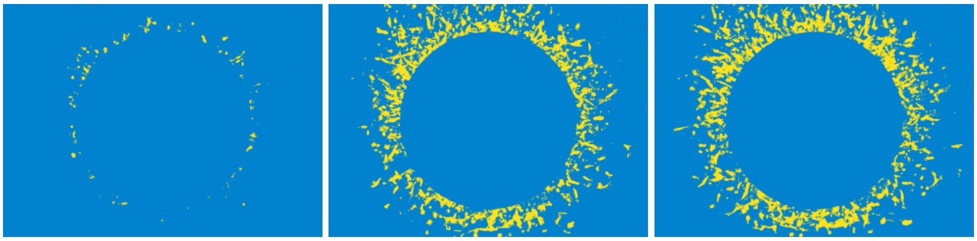

## 2. Workflow for GIMP, ImageMagick, Ilastik and CellProfiler

Step 1: In GIMP draw a circle that matches the border of the well and register the position as in (x,y) coordinates

Original image
1596x 1433 (Pixels)

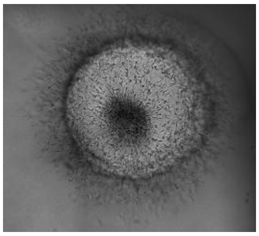

Mask

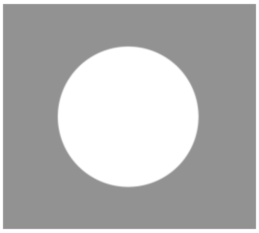

Step 2: In Imagemagick create a circular mask from the (x,y) coordinates obtained in GIMP.

draw circle\\ magick convert -size 1500x1400 xc:none -fill white -draw "circle 804,724 824,1094" mask.png

combine mask and image\\ FOR %i IN (*.tif) DO magick composite mask.png %i -matte %i

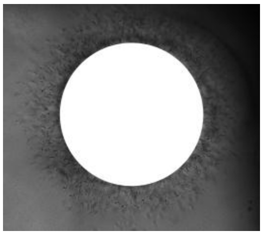

crop image to remove white\\ FOR %i IN (*.tif) DO magick convert %i -crop 1600x1496 %i

Step 2: A crop to divide the image in 4 equal parts is needed. The idea is that the white part of the image (originally the well) should be exactly in the middle of the image and equally distant from the borders)

crop image for 4 parts\\ FOR %i IN (*.tif) DO magick convert %i -crop 1538x1142+0+354 %i

remove second layer of tif file (detrimental for ilastik)\\ FOR %i IN (*.tif) DO magick convert %i[0] %i

Step 2: In Illastik run the previously trained pixel classifier.

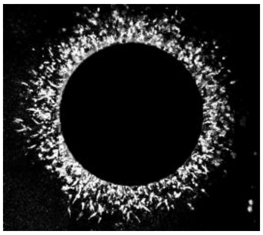

Probability map for Class 0

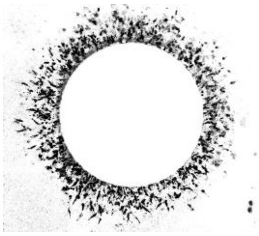

Probability map for Class 1

Step 1: Now it is required to divide each image in four quadrants of equal size. In this exemple the image here displayed has a width of 1538 and a hieght of 1118. To get 4 quadrants we need to obtain 4 parts of (11538/2 and 118/2) 769 width and 559 hieight.

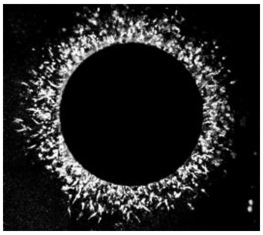

divide images in 4 quadrants\\ FOR %i IN (*.tif) DO magick convert %i -crop 769x559 %i-part%d.tif

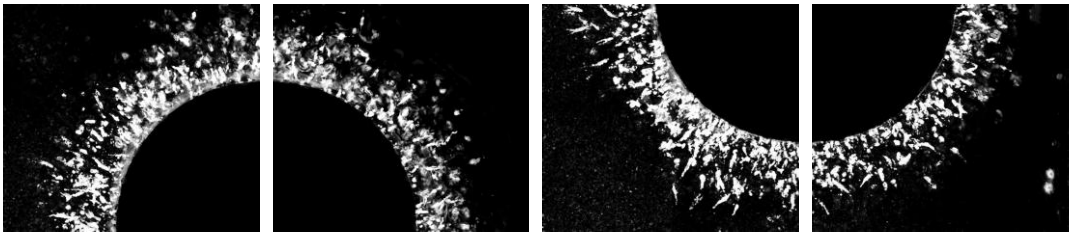

part0 part1 part2 part3

Load Images in CellProfiler

Identify Primary Objects
Thresholding strategy: Global
Thresholding method: Manual
Manual Threshold: 0.5

Measure Image Area Occupied

Display Data on Image

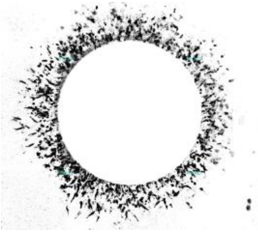

Export to Spreadsheet
